# Recent Progress in Optical Sensors for Biomedical Diagnostics

**DOI:** 10.3390/mi11040356

**Published:** 2020-03-30

**Authors:** Muqsit Pirzada, Zeynep Altintas

**Affiliations:** Institute of Chemistry, Technical University of Berlin, Straße des 17. Juni 124, 10623 Berlin, Germany; muqsit.pirzada@campus.tu-berlin.de

**Keywords:** optical sensors, biomedical diagnostics, surface plasmon resonance, evanescent wave, bioluminescence, ellipsometry, surface enhanced Raman scattering, reflectometric interference spectroscopy, biosensors

## Abstract

In recent years, several types of optical sensors have been probed for their aptitude in healthcare biosensing, making their applications in biomedical diagnostics a rapidly evolving subject. Optical sensors show versatility amongst different receptor types and even permit the integration of different detection mechanisms. Such conjugated sensing platforms facilitate the exploitation of their neoteric synergistic characteristics for sensor fabrication. This paper covers nearly 250 research articles since 2016 representing the emerging interest in rapid, reproducible and ultrasensitive assays in clinical analysis. Therefore, we present an elaborate review of biomedical diagnostics with the help of optical sensors working on varied principles such as surface plasmon resonance, localised surface plasmon resonance, evanescent wave fluorescence, bioluminescence and several others. These sensors are capable of investigating toxins, proteins, pathogens, disease biomarkers and whole cells in varied sensing media ranging from water to buffer to more complex environments such as serum, blood or urine. Hence, the recent trends discussed in this review hold enormous potential for the widespread use of optical sensors in early-stage disease prediction and point-of-care testing devices.

## 1. Introduction

Optical sensors constitute the most widely used class of biosensors as they possess several merits over conventional analytical techniques. They allow real-time, rapid, cost-effective and label-free detection of several different analytes and are highly specific, sensitive and small in size [1]. Novel sensors are products of multiple highly interdisciplinary areas such as chemistry, molecular biology, microelectromechanical systems, nanotechnology and micro-electronics [2]. Recent developments in the fields of proteomics, genomics and biology have enabled a better understanding of the binding kinetics, dissociation-association rates, analyte-ligand interactions and cross reactivity of the sensor with non-specific molecules [3]. As the research and development in optical sensors has seen an exponential growth in the previous decade, such sensors present enormous potential for applications in the biotechnological industry, ecological sciences and healthcare [1]. The detection time, reusability, output precision and cleaning requirements of optical sensors can be tailored according to the concentration and characteristics of the analyte [4].

**Figure 1:**
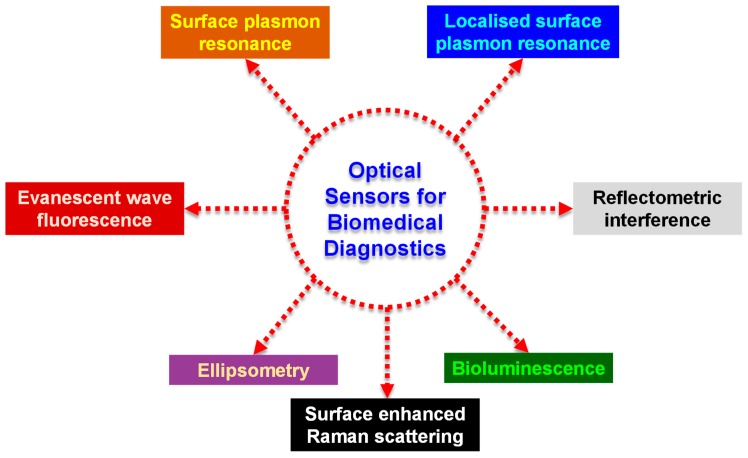
Different types of sensors classified on the basis of the underlying optical phenomenon arising from receptor-analyte interactions.

Optical detection is more common than electrochemical or piezoelectric techniques and it is performed by harnessing the optical field with a moiety capable of biorecognition [1,3]. Optical sensors can be classified on the basis of their detection modes (label-based or label-free), recognition mechanism or the optical phenomenon exploited for sensing [5]. Although the introduction of a label is an additional step, any desired wavelength can be used for the analysis in label-based sensors [6,7]. Relying on the type of recognition mechanism, optical biosensors can be classified into catalytic and affinity-based sensors. In case of catalytic optical sensors, biochemical species are recognised and converted into products *via* a chemical reaction by the biocomponent. Affinity-based sensors make use of the specific abilities of the receptor to bind with the analyte [3,5,6]. Based on the phenomenon exploited for sensing the receptor-analyte interactions, Figure 1 shows the different varieties of sensors used for health care diagnosis.

**Figure 2:**
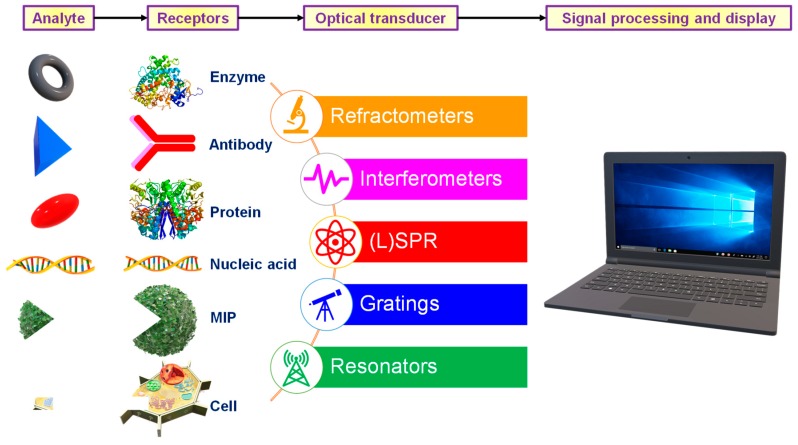
Construction of different optical sensors.

Sensors comprise of a receptor responsible for interacting with the analyte, a transducer that produces a signal in proportion with the concentration of the analyte and a detection system which enables the quantification of the signal into usable information [2]. They are classified based on the signal transducer into electrochemical [8], thermometric [9], piezoelectric [10], magnetic [11] or optical [12]. Optical sensors are compact devices capable of analysis using a receptor and an optical transducer [13]. The primary goal of such a system is to produce optical signals commensurate with the concentration of the analyte in the sensing medium [1]. The receptor may be of natural (antibody [14], protein [15], nucleic acid [16]), semi-synthetic (peptide [17]) or synthetic (molecularly imprinted polymers [18]) origin. Surface plasmon resonance (SPR), localised surface plasmon resonance (LSPR), optical waveguide interferometry and evanescent wave fluorescence exploit the evanescent region close to the sensor surface to investigate the interaction between the analyte and the receptor [1]. A large variety of optical sensors with different constructions can be fabricated as illustrated in Figure 2. This review focuses on a few of the most widely used optical detection systems for healthcare biosensing applications.

## 2. Surface Plasmon Resonance Sensors for Biomedical Diagnostics 

SPR sensors operate on a facile phenomenon comprising of five events [19]: (1) The electromagnetic field arising from the interactions at a metal-dielectric interface excites the combined coherent oscillations belonging to the free electrons situated in the conduction band of the metal. (2) This excitation generates oscillations in the charge density known as surface plasmon polaritons (SPPs). (3) The SPPs create an exponentially decaying electric field in the ambient medium wherein the penetration depth ranges in between a few hundred nanometres. (4) This resultant evanescent field shows extreme sensitivity towards deviations in the refractive-index of the ambient medium. (5) During resonance, the incident light beam is absorbed at a particular wavelength or incident angle which results in an excitation peak in the signal measured signal spectrum and a shift in this peak occurs due to variations in the refractive-index of the sensing medium owing to the presence of the analyte. One of the most common types of SPR sensors relies on propagating surface plasmon resonance (PSPR). Generally, PSPR is excited through grating or prism couplers on continuous thin metal films [19,20,21]. It can propagate in the sub-millimetre range [19]. PSPR sensors have superior sensitivities in comparison to localised surface plasmon resonance (LSPR) [22,23]. SPR sensors constitute the most widely researched type of optical sensors and have therefore garnered a lot of attention from the scientific community in recent years [1,24]. 

**Figure 3:**
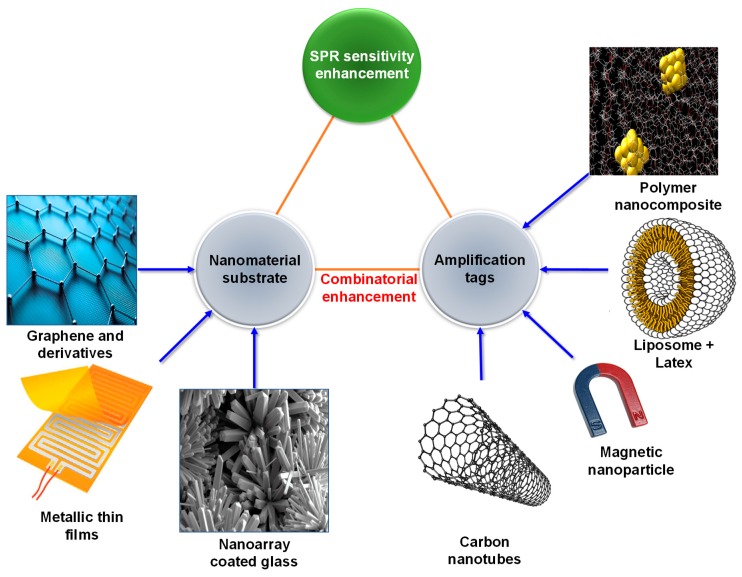
Various strategies to enhance the signal of SPR sensors.

Usually, it is challenging to sense extremely dilute concentrations (<1 pM) or low molecular weight compounds (< 8 kDa) using PSPR [19,22,25,26]. Therefore, the sensitivity of various SPR sensors needs to be enhanced frequently using techniques illustrated in Figure 3. Most of these strategies involve the use of nanomaterials such as gold, silver or other metallic nanoparticles, nanocages, nanorods, nanoarrays, nanostructured films, magnetic nanoparticles as well as graphene and its derivatives such as graphene oxide and reduced graphene oxide (GO) [20,22,27,28]. Noble metallic nanomaterials, particularly gold nanoparticles (AuNPs) enhance the SPR properties of the substrate and are also referred as plasmonic nanoparticles [20,22,29,30]. Plasmonic nanoparticles provide strong absorption of light in the near – infrared and visible regions resulting in a larger excitation of the electric field which causes a bigger field enhancement and consequently higher sensitivity [19]. Therefore, Wu et al. developed a nanohybrid of Fe_3_O_4_–hollow gold particles (HGNPs) with shell diameter of 44 ± 5 nm and 3 ± 0.6 nm thickness as a signal probe to obtain a magnetic-field assisted SPR-based sensor for the detection of human immunoglobulin G (IgG) [31]. The sensing platform comprised of a carboxyl functionalised graphene oxide film on a gold substrate. Immobilisation of capture antibodies (Ab_1_) on the graphene oxide film and detection antibody (Ab_2_) on the Fe_3_O_4_–HGNPs nanoconjugates resulted in a sandwich immunoassay with significant plasmonic fields as the magnetic nanoparticles increased the target – receptor binding within the active volume. In comparison to a conventional sandwich assay (500 ng mL^−1^), the nanoparticle probes amplified the signal considerably and reduced the limit of detection (LOD) 260 times (1.88 ng mL^−1^) [31]. The same group also reported a similar sensor in the following year for the detection of cardiac troponin I (cTnI) in which nanocomposite film of the same HGNPs and polydopamine (PDA) was deposited on a gold substrate [32]. The wavelength modulated SPR immunosensor utilised magnetised multi-walled carbon nanotubes (MMWCNTs) deposited with PDA. Non-binding sites of the capture antibody were blocked with bovine serum albumin (BSA). The plasmonic particles allowed facile extraction *via* the application of a magnetic field and also reduced the limit of detection 1000-fold to 1.25 ng mL^−1^ [32]. 

Wang and co-workers used a nanomaterial-based substrate for enhancing SPR signals to enable the Mach-Zehnder based screening of cetuximab, a vital drug used in the treatment of lung adenocarcinoma, colon cancer as well as breast cancer [33]. The sensor was developed on a 45 nm thick gold nanofilm with a negative resist based microfluidic channel. The differential interference-based phase sensitive SPR showed a high stability of 6 × 10^−7^ refractive index units (RIU) in 80 min. The high affinity (dissociation constant = 4.19 ± 0.58 nM) SPR sensor determined the cell sensitivity to the drug within 4 h [33]. In another research, a 56.3 nm thick AuNP film was adsorbed on an optical fibre prior to PDA deposition to generate an immunosensor for IgG detection [34]. The sensor showed no loss in performance over a ten-day period and could be completely regenerated on incubation in piranha solution (where H_2_SO_4_ : H_2_O_2_ ratio is 7:3) for just 1 minute. Neoteric SPR sensors may also be constructed by varying the shape of the plasmonic nanoparticles. For example, Heidarzadeh analysed haemoglobin concentration in blood using spherical (radius = 35 nm), cubic (edge = 45 nm) and cylindrical (radius = 35, height = 40 nm) plasmonic silver nanoparticles (AgNPs) to exploit the minor changes in refractive index arising from biomolecular interactions capable of shifting the sensitive SPR peaks [35]. Although all samples provided linear responses from 0–140 g mL^−1^, cylindrical AgNPs showed a noticeably higher wavelength shift (5.5 nm) in comparison to cubic (3.7 nm) and spherical particles (3.3 nm). 

**Figure 4:**
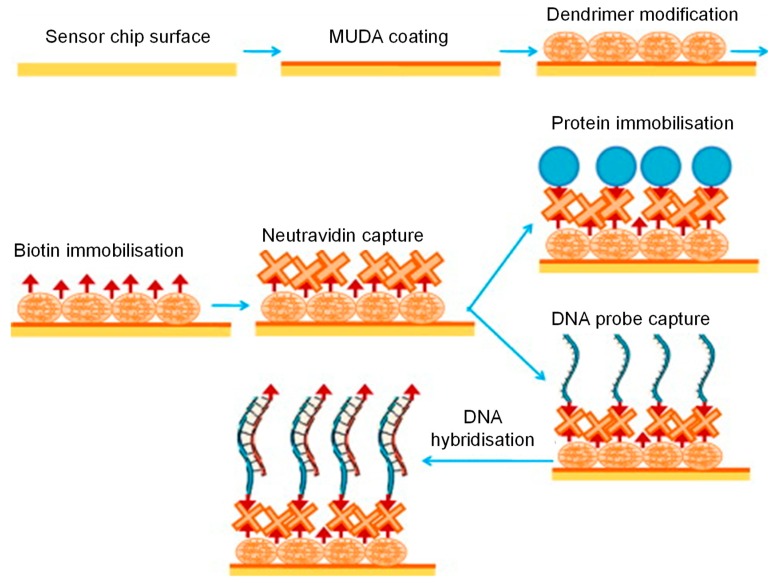
Surface functionalisation of a gold chip for SPR signal enhancement [36]. Reproduced with permission from [36].

Surface modifications, such as the ones shown in Figure 4, have also been widely explored to enhance SPR signals [36]. In a study that relied on a combinatorial approach for amplifying the SPR signals, gold nanostars (AuNSs) acted as amplification tags in a carboxyl-functionalised GO-based SPR sensor for the determination of IgG [37]. The AuNSs (core diameter = 27 nm, branch length = 12 nm, tip diameter = 9 nm) coupled with carboxyl functionalisation of the GO film (thickness = 0.963 nm) reduced the LOD 32 times in comparison to an unenhanced GO-based sensor. Another interesting strategy used in developing SPR sensors is the use of molecularly imprinted polymers (MIPs) as receptors. Altintas et al. studied monomer-analyte interactions and energy profile of the monomer complex with the help of computational simulations to synthesise rationally designed MIP nanoparticles (nanoMIPs) with diameters in the range of 200 to 235 nm [38]. Integration of *in silico* designing to sensor fabrication allows tremendous reduction in the research cost as well as experimental work and time. Such high-affinity nanoMIPs of varying particle diameters have been used for the detection of endotoxins (190–235 nm) [38,39], drugs (169.4 ± 3.5 nm) [40], viruses (205–238 nm) [41] as well as antibiotics (174 ± 2.16 nm) [42] with the help of SPR sensors.

**Figure 5:**
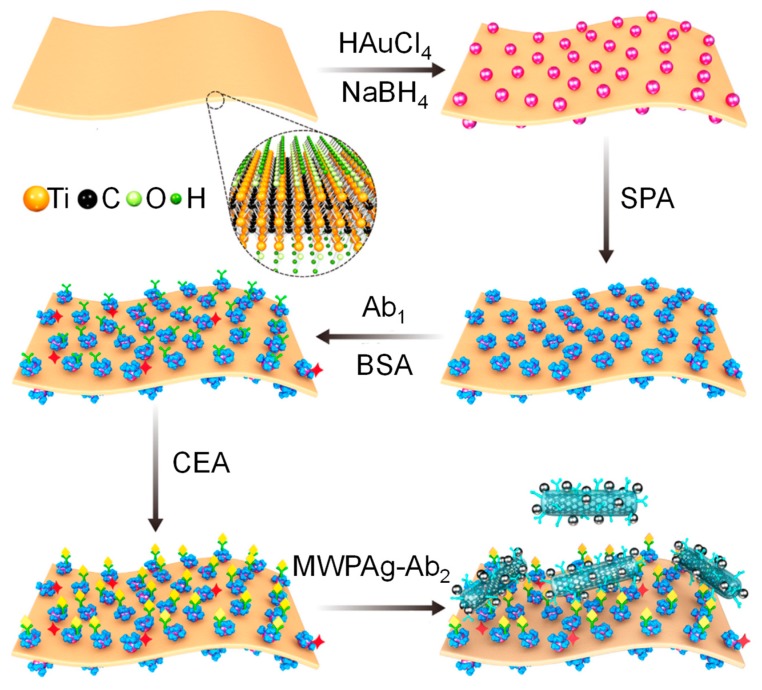
Construction of Ti_3_C_2_-MXene-based SPR sensor for CEA detection [71]. Reproduced with permission from [71].

SPR sensors have also been widely explored for early stage cancer diagnosis [43,44,45,46,47,48,49]. They have been used for the detection of prominent cancer biomarkers such as cytokeratins [50,51,52], prostate specific antigen (PSA) [53,54,55], nucleic acids [56] and several other such molecules [57,58,59,60,61,62]. For example, Chen’s group reported a label-free cytosensor for breast cancer diagnosis based on a mucin 1 aptamer immobilised gold substrate [63]. Nanoconjugates of folic acid and monodisperse magnetic nanoparticles (MNPs) acted as additional selective binding reagent to allow the formation of an ultrasensitive sandwich assay. The high refractive index and large mass of MNPs with a particle diameter of 13 nm allowed the detection of Michigan cancer foundation-7 (MCF-7) cell line down to 500 cells mL^−1^ [63]. Another study on MCF-7 cytosensing relied on a calixarene crown ether functionalised gold chip (film roughness = 1.60 nm) to obtain a direct assay with a significantly lower LOD (197 cell mL^−1^) [64]. Eletxigerra et al. developed an SPR sensor for erythroblastosis oncogene B2 (ErbB2), another vital breast cancer biomarker [65]. ErbB2 detection in serum could be achieved for as low as 180 pg mL^−1^. The sensor developed by Narayan and co-workers consisted of a gold substrate with a self-assembled monolayer (SAM) of 11-mercaptoundecanoic acid [66]. The SAM acted as a stabile substrate for the perpendicular adsorption of monoclonal antibodies against endothelin-1, a biomarker for colorectal cancer. The sensor showed high sensitivity (2.18 m^o^ pg^−1^ mL where m^o^ was the SPR angle) over a wide concentration range (2–100 pg mL^−1^) and low LOD (0.30 pg mL^−1^). The efficacy of the SPR sensor as a point-of-care testing (POCT) device was also established using electrochemical methods. Furthermore, the sensor showed a performance similar to enzyme-linked immunosorbent assay (ELISA) in serum [66]. Rebelo et al. developed a MIP-based sensor capable of detecting carbohydrate antigen 125 (CA-125), a biomarker for several types of cancer, *via* optical (SPR) as well as electrochemical (square wave voltammetry) techniques [67]. The imprinting process involved SAM formation of cysteamine on a screen-printed gold electrode that encouraged CA-125 adsorption. Subsequently, pyrrole was polymerised into a thin film and the template was finally removed by incubating the electrode in a sodium dodecyl sulfate solution. The sensor was capable of detecting over a wider concentration range with the electrochemical investigation strategy (0.01–500 U mL^−1^) in comparison to the SPR strategy (0.1–300 U mL^−1^) [67]. Nevertheless, the linear response range was far broader than the similarly developed surface plasmon resonance imaging immunosensor reported by Szymańska’s group, which was only 2.2–150 U mL^−1^ of CA-125 [68]. Although the MNP-based SPR sensor reported by Pal et al. provided a multiplex-based assay for the detection of CA-125 as well as other ovarian cancer biomarkers (β2-microglobulin and Apo-lipoprotein A1), the calibration curves revealed an even narrower linear response range for CA-125 (5.0–40.0 U mL^−1^) [69] than the former two studies [67,68]. Thus, a comparison between the three studies established the superiority of molecular imprinting over antibody-based receptors. This trend was also observed when the sensing buffer was replaced with serum. Carcinoembryogenic antigen (CEA) is another prominent cancer biomarker explored using SPR sensors [70,71,72]. Li’s group established the merits of streptavidin modified 10 nm large AuNPs as signal enhancers for SPR-based CEA detection by comparing the performance of a direct assay, a sandwich assay without AuNPs and streptavidin functionalised AuNPs enhances sandwich immunoassay [72]. The LOD reduced 4.2-fold when the direct format was replaced with the sandwich format and 13.8-fold when the sandwich assay also included the modified AuNPs. Wu et al. also reported an SPR sensor for CEA determination utilising MXene, a neoteric material consisting of layers of transition metal nitrides, carbides, carbonitrides, *etc* which has a thickness of a few atoms [71]. As illustrated in Figure 5, the device consisted of ultrathin Ti_3_C_2_-MXene (thickness = 2 nm) nanosheets which served as a binding substrate for AuNPs (size = 40 nm). This nanohybrid was further functionalised with staphylococcal protein A (SPA) which helped control the orientation and immobilisation of monoclonal capture antibodies (Ab_1_). The label consisted of polyclonal detection antibodies (Ab_2_) immobilised on a nanocomposite of multi-walled carbon nanotubes (MWCNT), PDA and AgNPs (MWPAg) with mean particle size of 30 nm. The sensor provided a linear response against the logarithm of CEA concentration from 200 aM to 20 nM and the LOD was 70 aM [71] which showed a higher sensitivity than several contemporary SPR-based CEA sensors [73,74].

SPR sensors have also been used in diverse healthcare biosensing applications, bioimaging paediatric burns [75] as well as in the study of mutations [76] and the effects of radiation exposure [77]. Recent studies have also shown the versatility of such devices in the study of pathogens [78], thermal injuries [79], carcinogens [80] and several clinically vital molecules [81,82,83].

## 3. Localised Surface Plasmon Resonance Sensors for Biomedical Diagnostics 

**Figure 6:**
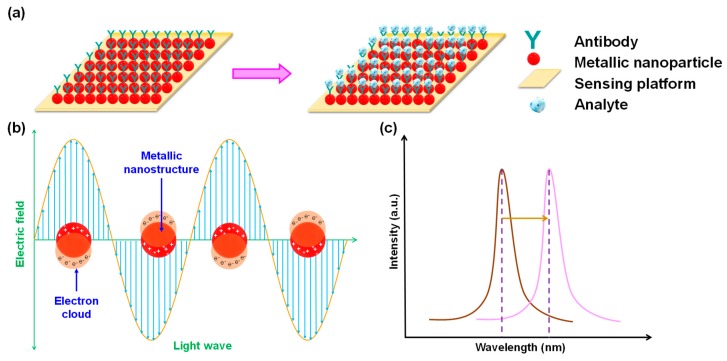
(**a**) LSPR-based direct assay; (**b**) Principle of LSPR; (**c**) Shifting of the LSPR extinction peak to higher wavelength from an analyte free state (brown curve) to analyte bound state (pink curve).

Widespread application of PSPR in the detection of very small analytes (few nanometres in size) is inhibited by the several hundred-nanometre thick plasmon modes of the metallic films employed. Thus, LSPR presents a viable alternative to the elaborate hydrogel-based functionalisation strategies necessary for small molecule recognition using PSPR [84]. Unlike PSPR where the surface plasmons arise due to interaction of incident light with thin metal films, the electromagnetic waves interact with conduction electrons of metallic nanocrystals resulting in LSPR (Figure 6a). As metals possess abundant electrons in the conduction band, the electrons oscillate collectively due to excitation from the incoming photons [85]. These oscillations within the nanoscale spatial confinements generate prominent resonance bands in scattering as well as absorption (Figure 6b) [86]. The typical optical response of such nanostructures is the result of several intrinsic parameters such as the material, dimension, geometry, and shape. Furthermore, extrinsic influences such as the change in the refractive index due to biomolecular interactions in the ambient media are pivotal to LSPR-based sensing. Figure 6c illustrates the shift in the spectral peak, arising from scattering or extinction, to a larger wavelength. In addition, enhancement within the near field due to such plasmonic nanostructures gives rise to non-elastic optical phenomena which are very important to develop sensors based on surface-enhanced Raman spectroscopy (SERS) [85,87,88,89,90]. Although the highest scattering efficiency is exhibited by silver, it can undergo surface oxidation on atmospheric exposure which can prove deleterious to plasmonic resonance in biosensing. Hence, AgNPs are subjected to functionalisation prior to their application in sensor development [91]. Due to higher stability as well as a superior capability to undergo functionalisation with various biomolecules, AuNPs are emerging as the material of choice to develop LSPR transducers [84,92,93] even though the magnitude of their relative scattering efficiency is lower than that of AgNPs [86]. Although aluminum nanoparticles display an analogous oxidation behavior to AgNPs, it is emerging as an important material for transduction in LSPR because of lucrative preparation strategies that allow spectral tunability within the ultraviolet (UV)/ visible (vis) range [59,86,94,95].

The performance of LSPR sensors critically depends on the shape of the nanoparticle used. Higher sensitivities and local electromagnetic fields were reported for nanostructures with anisotropic geometries in comparison to spherical nanoparticles [86]. Nanorods, with high aspect ratios, constitute one of the most widely investigated materials for the fabrication of LSPR transducers. Unmodified gold nanorods (AuNRs) of 50 nm length have been used for deoxyribonucleic acid (DNA) detection down to 1.47 nM with a colorimetric sensor [96]. Wang et al. studied telomerase activity in as low as 15 HeLa cells using the AuNR (70 nm length in absence of HeLa cells) etching strategy [97]. Another group also reported a similar technique for the ultrasensitive detection of inorganic pyrophosphatase activity [98]. AuNR functionalisation with nucleic acids [99,100] as well as antibodies [93,101,102] has been widely reported. Kong’s group developed a neoteric and cost-efficient sensor for the detection of bleomycin, a drug commonly administered in the treatment of lymphoma, ovarian as well as testicular cancer [103]. The sensor was fabricated using a composite of tungsten sulphide nanorod array on titanium mesh with DNA functionalised AuNPs (size = 18 ± 1 nm) as a sensor platform and a multiplexed signal amplifier in the form of a nanohybrid of AgNPs (lattice spacing = 0.27 nm) and zinc metal-organic framework nanozyme. Other geometries that have been widely explored in recent years for developing LSPR sensors include nanopyramids [95,104,105], nanourchins [106,107,108,109], nanocups [92,110,111,112,113], nanoholes [114,115,116,117] and nanoislands [118,119,120,121,122]. 

Although an excellent LSPR signal comprises of a sharp bandwidth with high-intensity resonance peak, performance enhancement proves insufficient. This is because the thickness of nanofilms is very low to curb the exponential decay of LSPR [86]. Therefore, optimisation of the LOD of LSPR biosensors demands signal enhancement. This can be achieved *via* two strategies: (1) local electromagnetic field enhancement; (2) resonance coupling. The former strategy involves the introduction of shape anisotropy as well as the effectuation of hotspots on and around the nanostructures. This technique has been used by different types of Raman spectroscopies, surface-enhanced infrared absorption spectroscopy, metal-enhanced fluorescence and metal-quenched fluorescence [86,123,124,125]. The latter strategy relies on the interactions between the plasmonic nanostructures from the transducer with other plasmonic particles giving rise to plasmon hybridisation and is often employed in colorimetry, fano-resonance, Förster resonance energy transfer as well as molecular plasmonic switching [126]. Fano-resonance arises from the destructive interference of the isolated resonant scattering phenomenon with the continuous propagation mode from the background resulting in an asymmetric line shape [127]. Fano-resonance biosensors are suitable for detecting a variety of analytes due to significant changes in the frequency spectrum as well as the intensity of transmission due to minor perturbances in the ambient optical properties [128,129]. They are emerging as model materials for developing lab-on-a-chip biosensors [130] and have demonstrated a 2000 fold enhancement in the sensitivity over conventional SPR sensors [131]. 

LSPR sensors can be constructed using various types of receptors such as aptamers [55,132,133], antibodies [119,134,135,136,137,138,139,140], MIPs [141,142,143,144], and nucleic acids [100,145,146]. The combination of LSPR with optical fibres to fabricate ultrasensitive sensors for cancer biomarker diagnosis has transformed into an evolving trend. Fibre-optic LSPR biosensors [54,139] show a nearly 1000 fold higher sensitivity than LSPR sensors using aptamer functionalised gold nanodisk arrays (diameter= 100 nm, thickness = 20 nm, spacing = 200 to 30 nm) [55]. The sensitivity of virus detection has been reported to improve by seven orders of magnitude when AuNP (diameter = 26.5 ± 0.5 nm)–quantum dot (QD) nanoconjugates [147] are preferred over AgNPs (diameter = 20–80 nm) [148] as plasmonic nanomaterials owing to the oxidative degradation of AgNPs. Although signal enhancement of AuNPs for virus detection is lower in the case of nanohybrids using CdSeTeS QDs (size = 10.1 ± 2.9 nm) [137,149] or CdZnSeS QDs (size = 4.8 ± 0.6 nm) [147], a 33-fold lower LOD was reported when CdSeS QDs (size = 2.7–7.8 nm) were functionalised with a molecular beacon to obtain hairpin hybridisation assays [146]. Li’s group established the superiority of LSPR over electrochemical sensing in the study of several neurotransmitters [117]. Furthermore, Amiri et al. added carbon dots to the dopamine sensing system (nanoconjugate diameter = 90.0 nm) to enable a 100-fold higher sensitivity [150]. It was reported that the detection limit for dopamine can vary inversely with the aspect ratio of plasmonic nanoparticles [151,152]. LSPR sensors allow the bioanalysis of different kinds of biomarkers that facilitate the diagnosis of cancer [153], pathogens [146,154], auto-immune diseases and immunodiagnostics [86,155] and constitute an important class of biosensors that are capable of replacing the conventional SPR-based detection strategies [84]. 

## 4. Evanescent Wave-Based Sensors for Biomedical Diagnostics 

**Figure 7:**
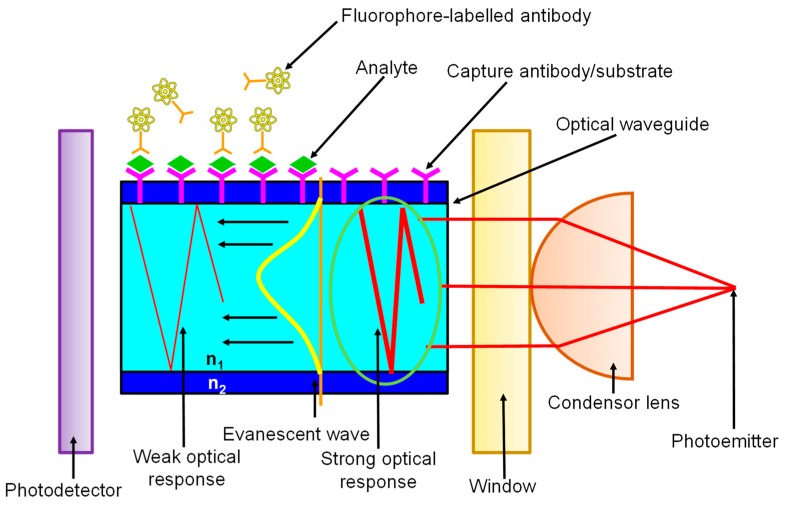
A schematic illustration of evanescent wave-based sensing (*n_2_* <*n_1_*).

In evanescent wave-based sensors, the analyte-receptor interaction and subsequent recognition take place within the confines of an evanescent wave (EW). The EW is a result of the behaviour of light when its propagation is restricted within an optical fibre or waveguide [1]. Figure 7 shows that as the guided light propagates, it undergoes total internal reflection (TIR) at the interface of the waveguide and the surrounding medium of lower refractive index (*n*_2_ < *n*_1_). Since the Fresnel coefficients of the transverse magnetic and electric waves for TIR are non-zero, an electromagnetic field arises at the interface and extends into the medium with a lower refractive index despite the total reflection of light energy. This field undergoes an exponential decay with distance from the surface, usually over a distance of one wavelength or approximately 100 nm [156] making EW a near-surface phenomenon. EW fluorescence biosensors rely on measurements in which the surface fluorophores are selectively excited. Such geometric confinement facilitates the preferential signal enhancement from the fluorophores on the surface while allowing the minimisation of undesirable background noise from the bulk sample [157]. The commercialisation of several waveguide-based sensors has spurred the development of a profuse variety of biosensors for several types of clinically relevant biomolecules [156].

**Figure 8:**
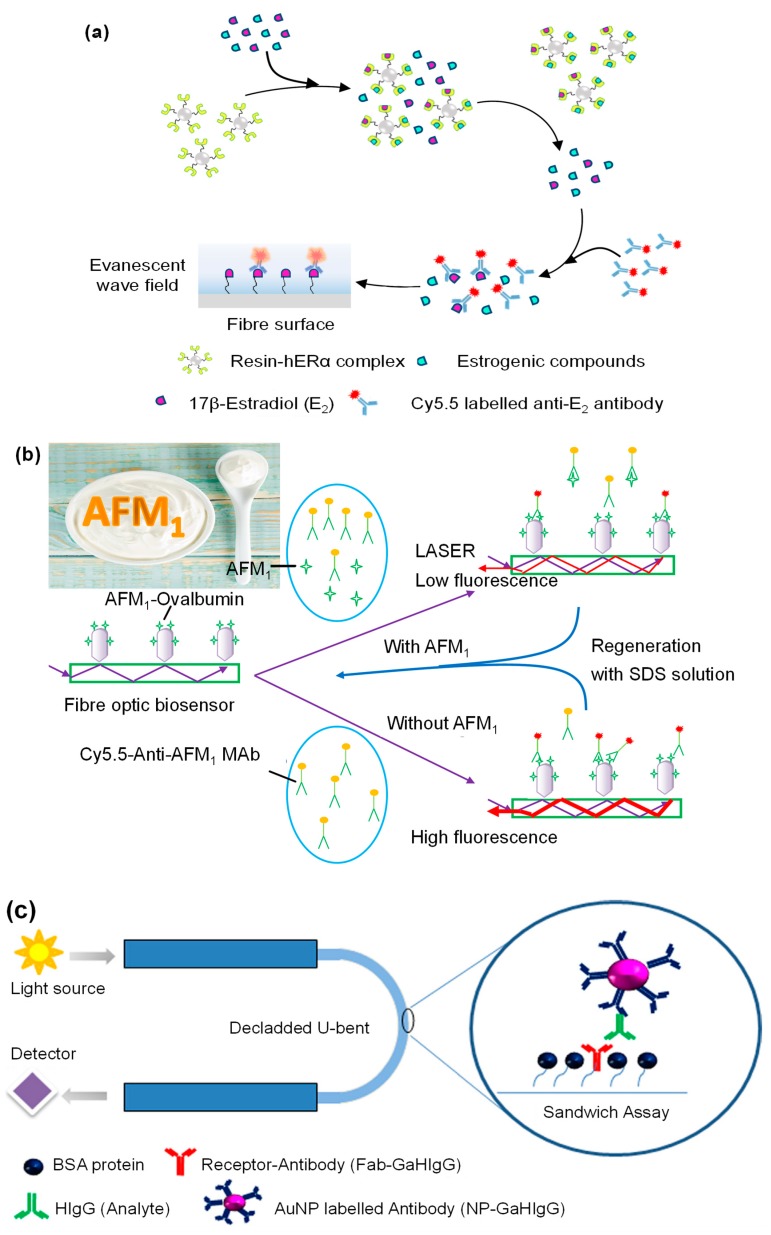

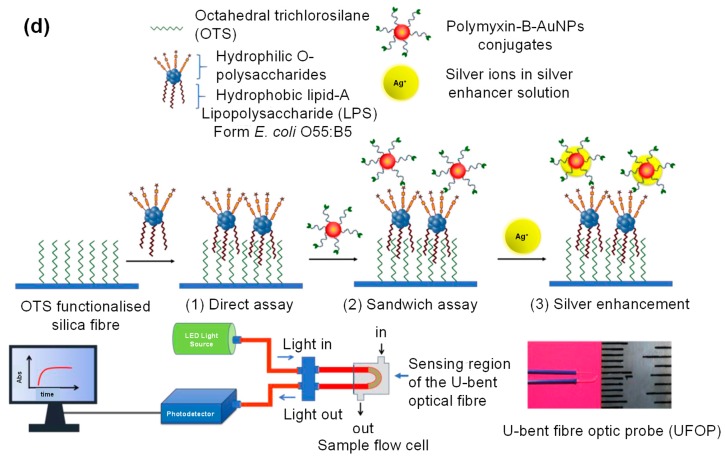
(**a**) Competitive binding assay for the detection of xenoestrogens [165]; (**b**) AFM_1_ recognition with an optofluidic platform [167]; (**c**) HIgG sandwich assay using a U-bent fibre probe [170]; (**d**) Bacterial endotoxin recognition and signal enhancement in a U-bent fibre optic probe [171]. Abbreviations: AFM_1_: Aflatoxin M_1_; Anti–AFM_1_: Aflatoxin M_1_ antibody; Anti-E_2_: 17β-estradiol antibody; AuNP: Gold nanoparticles; BSA: Bovine serum albumin; Cy5.5: Cyanine5.5; *E*. *coli*: *Escherichia coli*; Fab: Antigen binding fragment; hERα: human estrogen receptor α; GaHIgG: Goat antibody of human immunoglobulin G; HIgG: Human immunoglobulin G; LPS: Lipoploysaccharide; MAb: Monoclonal antibodies; OTS: Octadecyltrichlorosilane; SDS: Sodium dodecyl sulphate. Reproduced with permission from [165,167,170,171].

Evanescent sensors have been exploited for recognition of complex biomolecules as well as smaller analytes [158]. Optical fibres have been the substrate of choice to develop these sensors as they are compatible with several different classes of receptors. Split-aptamers have been investigated as receptors for the micromolar detection of adenosine using optical fibre substrates [159]. In comparison to conventional EW-based aptasensors [160], platforms using split aptamers [161] showed a 100-fold higher sensitivity and 2.6 times shorter assay time for small molecule detection. For larger and structurally complex molecules such as antibiotics, split-aptamers have not been probed as receptors but hold promise as even conventional aptasensors have allowed group-specific nanomolar detections [162]. Optical fibre-based sandwich immunosensors frequently employ AuNPs for signal enhancement which allows ultrasensitive and rapid detection [163,164]. Liu’s group developed an EW immunosensor using antibody labelled fluorophores for xenoestrogens which are molecules that mimic the structure of estrogen compounds and interfere with the functions of endogenous estrogens [165]. The sensor relied on a competitive binding assay of various xenoestrogens against 17 β-estradiol with the human estrogen receptor α (Figure 8a). The EW-based optical fibre platform showed more than 90% recovery in 300 sensing cycles [165]. Antibody receptors have also been coupled with optofluidic sensing systems and have shown high reusability without any loss of activity even after being used 100 times [166]. Such optofluidic immunoassays have also been developed for aflatoxin M_1_ (AFM_1_) and showed high sensitivity (LOD: 5 ng mL^−1^) after 200 cycles of regeneration (Figure 8b) [167]. However, substituting the optofluidic system with a multiplexed planar waveguide fluorescence system brought more than 100 fold increment in sensitivity of AFM_1_ (LOD: 45 pg mL^−1^) with recovery rates between 85 %–103 % indicating very high accuracy [168]. MIPs have been explored as synthetic receptors in EW-based sensing platforms. Liu and co-workers developed an optical micro-fibre interferometer with molecularly imprinted PDA receptor for detecting C-reactive protein (CRP). The sensor was capable of quantifying CRP down to 581.3 zg mL^−1^ which was 8 times lower than conventional sandwich ELISA [169].

An interesting strategy that is being adopted to enhance the sensitivity of EW sensors is the use of U-bent fibre optic probes with functionalised AuNPs (Figure 8c). Manoharan’s group used such a setup for the detection of bacterial endotoxins with the help of a Polymyxin-B labelled AuNPs [171]. The detection time was just one hour and the sensor showed a 36-fold sensitivity improvement upon silver enhancement (Figure 8d). Although such an assembly has been employed for the detection of immunoglobulin [170], plastic optical fibre-based sensors in conjunction with liposomal amplifiers show 10^6^ times higher sensitivity [172]. Such plastic optical fibres have also enabled rapid pathogen detection in 30 minutes over a wide concentration range (10^4^–10^8^ MPN mL^−1^) [173]. EW-based non-enzymatic glucose sensors have been developed using a polyphenylnoronic acid. However, such sensors are not suitable for *in* vivo applications due to their size [174]. For this type of applications, glucose oxidase functionalised micromodal optical fibres are more suitable as they measure only a few micrometres in size which is comparable to the size of a single cell [175]. A similar sensor, using phase-shifted microfibre Bragg gratings, has also been reported for ultrasensitive detection of cTnI [176]. In addition to optical fibres and planar waveguides, 1-dimensional photonic crystals are also emerging as the optically active substrate for EW sensing. For example, Rizzo et al. developed a Blotch surface wave-based label-free sensor for the detection of vascular endothelial growth factor, a well-known cancer biomarker [177]. The assay was complete within 30 minutes and the LOD in human serum was 3.5 ng mL^−1^. A neoteric approach for sensitivity enhancement employed by Akhtar’s group includes growing ZnO nanoflowers on a glass slide coated with silver nanofilm for ultrasensitive detection of insulin amyloid [178]. Sophisticated assemblies such as dual-colour TIR fluorescence detecting platform [179] as well as optical fibre-polymer waveguide-optical fibre sandwich [180] set up have facilitated small-molecule detection down to picomolar levels. 

## 5. Bioluminescence-Based Sensors for Biomedical Diagnostics

**Figure 9:**
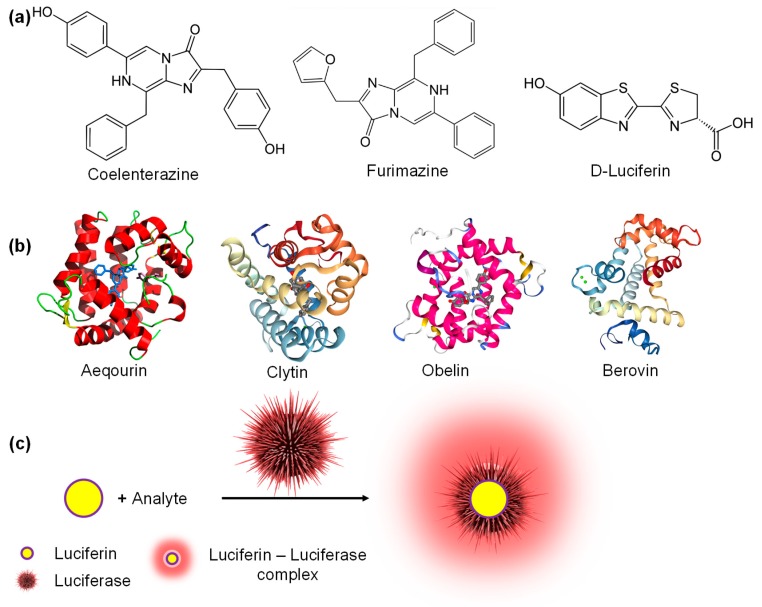
(**a**) Small molecules as luciferins; (**b**) Photoproteins; (**c**) Bioluminescence with a luciferin-luciferase assembly.

Bioluminescence is the production and emission of light by living organisms for defence, prey or communication [181]. The biochemical reaction involved in the production of light is the oxidation of a luciferin molecule (lucifer means “light-bearer”) by a luciferase enzyme [182]. Luciferins can be small molecules like coelenterazine, furimazine, D-luciferin (Figure 9a) with few cyclic structures as well as larger photoproteins such as aequorin, symplectin, pholasin, berovin, obelin and clytin (Figure 9b). The key difference between these two classes of luciferins is that photoproteins do not require enzymes for bioluminescence as light is emitted due to the development of charge on the surface of the protein in the presence of analyte molecules [181]. Fluorescence-based sensors require external light sources to function and are hence not generally suitable for studies involving live animals or thick tissues hindering widespread applications as POCT devices [183]. As the underlying principle for bioluminescence-based sensing is bioluminescence resonance energy transfer (BRET) which does not require external excitation, excited state-emitters can be generated with the help of enzyme-catalysed exothermic reactions (Figure 9c) [182].

**Figure 10:**
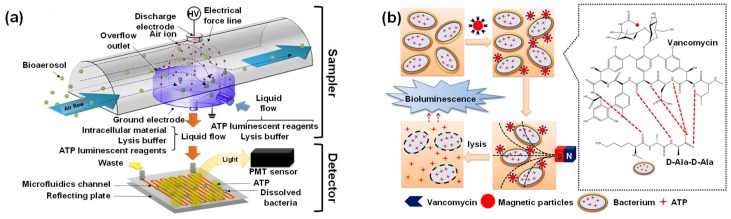

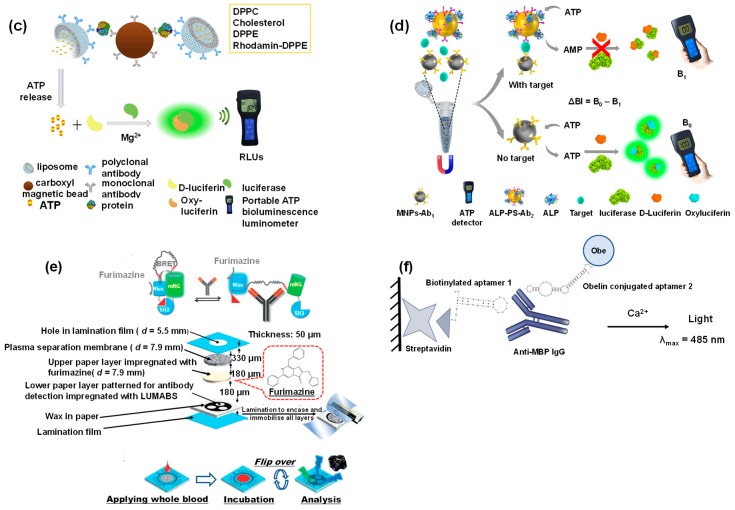
(**a**) BRET-based real-time bioaerosol monitoring device [186]; (**b**) Vancomycin conjugated magnetic particles for gram-positive bacteria detection [187]; (**c**) Magnetic nanoliposomes for bioluminescent protein sensing [196]; (**d**) Magnetic sandwich assay for procalcitonin detection [197]; (**e**) Furimazine-based antibody detection using LUMABS [202]; (**f**) Obelin as a photoprotein in the sensing of anti-myelin basic protein autoantibody [210]. Abbreviations: Ab: Antibody; Ala: Alanine; ALP: Alkaline phosphatase; AMP: Adenosine monophosphate; ATP: Adenosine triphosphate; B_0_/B_1_: Bioluminescence intensity; DPPC: 1,2-Dipalmitoyl-sn-glycero-3-phosphocholine; DPPE: 1,2-Dipalmitoyl-sn-glycero-3-phosphoethanolamine; IgG: Immunoglobulin G; MBP: Myelin basic protein; MNP: Magnetic nanoparticles; PMT: Photomultiplier tube; PS: Carboxyl modified polystyrene microsphere; Obe: Obelin. Reproduced with permission from [186,187,196,197,202,210].

Bioluminescent spectroscopy has a short detection time coupled with high detectability. These characteristics enable the longitudinal study of biological phenomena in diagnostic applications. Therefore, bioluminescence can be utilised for developing specific, selective and ultrasensitive cell-based as well as binding assays [181]. The most common luciferin employed in bioluminescence-based sensors is D-luciferin from fireflies. For example, Santangelo et al. developed a continuous flow monitoring system on a 3 D printed chip for adenosine triphosphate (ATP) detection [184]. With the help of silicon photomultipliers, this system was capable of nanomolar detection within 4 sec. The sensitivity of bioluminescent systems to ATP allows the monitoring of several different biological processes since ATP acts as an energy currency in living organisms. Therefore, Mondal’s group investigated the adenosine monophosphate (AMP) concentration to correlate the presence of ubiquitin down to 7 nM [185]. This luciferase-luciferin BRET system was capable of deconvoluting inhibitor specificity. Park and coworkers reported an interesting application of ATP-based biosensing which can be used in the development of biologic alarms [186]. They tested a bioaerosol system spiked with *Staphylococcus epidermis* in an indoor environment (Figure 10a) and the sensor exhibited a sensitivity of 1.66 CFU mL^−1^. Such BRET-based sensors are capable of rapid pathogen detection (Figure 10b) [187] and can be as much as 30 times cheaper than contemporary assays for bacteria detection [188]. Paper-based sensors have also been recently explored for the recognition of *E*. *coli* and although paper discs [189] show sensitivity which is an order of magnitude higher than lyophilised nanolanterns [190], the latter approach requires 10 times lesser reagents and is therefore significantly more lucrative. Mirasoli et al. coupled a loop-mediated isothermal amplification strategy with the bioluminescent assay in real-time for the specific detection of porovirus DNA [191]. The lab-on-a-chip sensor was equipped with temperature and radiation sensors and was capable of detecting 10 copies reaction ^−1^ in 20 minutes. Nucleic acid-based BRET sensors allow label-free picomolar detection of DNA, DNAzymes and other biomolecules with zero background signal [192]. Engineering the luciferase enzyme has broadened the scope of BRET sensors. For example, Mano and coworkers, fused the binding domain of the vitamin D receptor with a split luciferase to screen high-affinity ligands which can cause R274L mutation resulting in vitamin D resistant rickets [193]. An emerging trend in modern bioluminescent assays is the use of smartphones for facile analyte detection. Such systems have been employed in the generation of whole-cell toxicity assays [194] as well as in the detection of various disease biomarkers. Recently, a smartphone-based BRET sensor was used for the recognition of tumour necrosis factor α (TNFα). The novel biosensor comprised of a 3D spheroids with two different luciferases to enable the simultaneous investigation of inflammation and viability [195]. The introduction of nanomaterials into the assay enhances the signal and can even simplify the assay procedure. Magnetic nanoparticles allow ultrasensitive protein detection [196] with LODs that are 100 times lower than ELISA [197] (Figure 10c,d). Chen’s group conjugated a recombinant protein that contained Renilla luciferase with AuNPs to fabricate a thrombin sensor that allowed sensitive detection (LOD: 80 pM) in urine within a short time (10 min) [198]. Several derivatives of D-luciferin find application in biomedical diagnostics. A hydrazine D-luciferin system was capable of *in vitro* Cu^2+^ detection down to 39 nM and held promise for bioimaging applications [199]. Other vital derivatives include D-luciferin methyl ester [200] and Z-WEHD aminoluciferin [201]. Another small molecule that has attracted attention in recent years as a luciferin is furimazine. It has been used to develop microfluidic paper-based assays for simultaneous nanomolar antibody detection in whole blood (Figure 10e) [202] using a novel platform called LUMinescent AntiBody Sensor (LUMABS) [203]. LUMABS with furimazine substrates compares well against ELISA [204] and is compatible with several different antibodies [205]. Furimazine systems have also been employed for intracellular ATP sensing [206], voltage indication in live cells [207] as well as metal ion detection and bioimaging [207,208,209].

Apart from small molecules, photoproteins are also widely used to develop BRET sensors. One of the most common proteins that have gained increasing interest is green fluorescent protein (GFP). Hamer et al. established the superiority of BRET over FRET with the help of a GFP-based assay for monitoring caspase activity [211]. GFPs enabled a 48-fold higher sensitivity [212] for plasmin detection in comparison to a BRET^6^ assembly (red-shifted Renilla luciferase 8.6 with a fluorescent protein TurboFP635) [213] within the same assay time (10 min). UnaG, a basic GFP, has been used in conjunction with magnetic nanoparticle decorated silica_core_ poly(guanidine)_shell_ particles to make durable high affinity sensors for bilirubin (K_d_: 98 pM) [214]. Aequorin, a photoprotein, was recently used in conjunction with hybridoma technology for the first time in a proof-of-concept research on *Vibrio cholerae* diagnosis. The sensor allowed bacteria detection down to 50 CFU mL^−1^ within 7 seconds [215]. Krasitskaya’s group recently developed a dual-aptamer solid-phase sandwich-type microassay in serum for the ultrasensitive detection (LOD: 6.3 nM) of autoantibodies to myelin basic protein, a biomarker for multiple sclerosis, using obelin as the photoprotein (Figure 10f) [210].

## 6. Miscellaneous Optical Sensors for Biomedical Diagnostics

Although most of the progress in optical biosensors focused on SPR, LSPR, EW and BRET-based sensors, few other techniques such as ellipsometry, surface enhanced Raman scattering and reflectometric interference have recently emerged as interesting strategies for the detection of clinically relevant molecules. We have reviewed the biomedical application of these optical sensors in this section as comparatively fewer scientific works have been published in this arena so far. 

### 6.1. Ellipsometric Biosensors

Ellipsometry is a useful technique for studying dielectric characteristics of thin films [216]. Ellipsometric biosensors analyse the change in the elliptical polarisation of light due to transmission or reflection from the film surface and establish a comparison with a model [1]. As the incident radiation is extremely focused, analysis for very small sample sizes is also feasible with this technique. Interest in ellipsometric biosensors is increasing as it is the most surface sensitive optical diagnostic tool for film characterisation. In recent years, such sensors have been explored for the determination of micro-organisms, nucleic acids, proteins and several such biomolecules [217]. Nabok et al. combined total internal reflection ellipsometry (TIRE) with LSPR to detect as low as 10 ppt of mycotoxins with the help of a planar optical waveguide [218]. A similar approach was employed in the study of aflatoxin B_1_ [219]. Recently, TIRE has also been coupled with SPR for human immunodeficincy virus diagnostics [220], mycotoxin recognition [221] as well as micromolar immunoglobulin determination [222]. TIRE has also been explored for applications in bioimaging with the help of AuNPs [223]. Caglayan and Üstündağ developed an aptamer assay using attenuated internal reflection ellipsometry for the detection of zearalenone, a mycotoxin, down to 80 pg mL^−1^ [224]. Recently, there has been a rising interest in modifying conventional ellipsometric techniques to study vital biological processes. Kalas et al. investigated protein adsorption with the help of plasmonic ellipsometry with a Kretschmann-Raether geometry [225]. Similarly, Sohrabi and Hamidi utilised plasmonic ellipsometry to study brain activity [226]. In another study, non-linear optical Stokes ellipsometry was used for imaging the non-linear susceptibility tensor of collagen [227]. Ellipsometric sensors have also been fabricated in conjunction with solution immersed silica immunosensors for the rapid (5 min) determination of cTnI in blood serum [228].

### 6.2. SERS Biosensors

**Figure 11:**
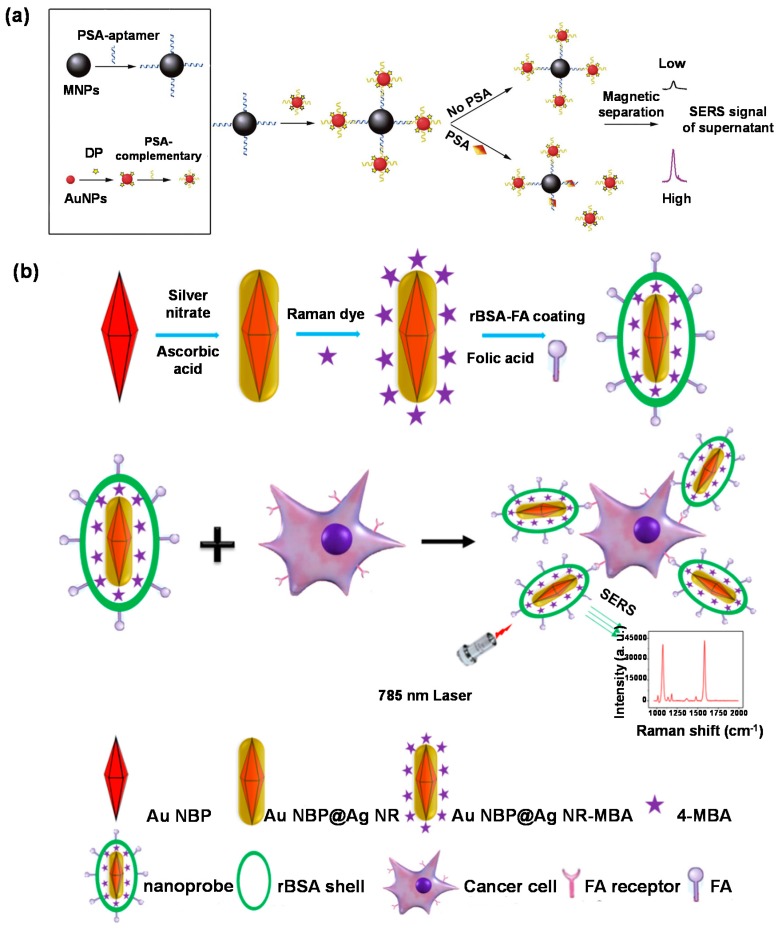
(**a**) Sandwich-type aptasensor for PSA detection with MNPs and AuNPs [233]; (**b**) SERS-based bioimaging of cancer cells [236]. Abbreviations: Ag NR: Silver nanorods; Au NBP: Gold nanobipyramid; AuNP: Gold nanoparticle; DP: 4,4’-dipyridyl; FA: Folic acid; MBA: 4-mercaptobenzoic acid; rBSA: Reduced bovine serum albumin; SERS: Surface enhanced Raman scattering. Reproduced with permission from [233,236].

The inelastic scattering of photons due to their interaction with matter is termed as Raman scattering. It is an extremely weak phenomenon with 6 to 10 orders of magnitude lower efficacy than fluorescence [229,230]. The scattering intensity can, however, be significantly amplified when the molecules involved are located in the vicinity of the surface of appropriate nanostructured substrates. This amplification modality is termed as surface-enhanced Raman scattering [231]. Considerable research in the field of SERS-based diagnosis has revolved around the optimisation of nanostructured substrates as the efficiency of the scattering signal is a function of the geometrical properties of the metallic nanoparticles [25,232]. Several SERS platforms have been reported for early-stage prostate cancer diagnosis. Some of the more researched morphologies include MNP–AuNP (Figure 11a) [233] and MNP-AgNP assemblies [234]. SERS-based genetic assays have been reported to be four orders of magnitude more sensitive than polymerase chain reaction (PCR) assays [235]. Such sensors were capable of bioimaging (Figure 11b) [236] as well as discriminating between healthy and cancerous cells [237]. 

SERS biosensors have also been developed for early diagnosis of colorectal [238] and breast cancer [239] as well as for monitoring the therapeutic effects during cancer treatment [240,241]. AuNPs [242,243] and AgNPs [244] have frequently been exploited in SERS-based hormone investigations. Functionalisation of Au_shell_–Ag_core_ with DNA enhanced the sensitivity of SERS assays for 17β–estradiol by 10-folds [245] in comparison to 4–mercaptobenzoic acid [246]. Innovative approaches such as conjugation of SERS with filter membranes make bacterial mapping feasible [247]. Core-shell assemblies have been used to obtain direct [248] as well as sandwich assays [249] for bacteria detection. Dual-enhancement using MNP_shell_–AuNP_core_ with vancomycin SERS tags exhibited a 3.3-fold higher sensitivity [250] than functionalised polymeric MNPs [251]. Optofluidic platforms allow ultrasensitive (LOD: 4 CFU mL^−1^) bacteria determination in a short assay time (15 min) [252]. SERS-based platforms hold enormous potential for the recognition of proteins [253,254,255,256] as well as for the identification cells [257] and genetic mutations [258].

### 6.3. Reflectometric Interference Spectroscopy Biosensors

Reflectometric interference spectroscopy (RIfS) is a time-resolved and label-free technique involving a lucid optical set-up involving white light interference at thin layers [259]. The analyte concentration is determined by variations in the amplitude and phase of polarised light due to changes in the refractive index and thickness of an adsorbed layer of the analyte [1]. Thus, the analyte binding phenomenon culminates in a shift of the pattern of interference within the wavelength domain [259]. Recently RIfS-based sensors were used for the detection of single nucleotides [260] as well as oligonucleotides [261] and they showed high durability to thermal and physical stress. Such sensors show a higher sensitivity than conventional lateral-flow systems for pathogen and toxin detection [262]. NanoMIPs have also been incorporated in RIfS platforms to fabricate highly stable sensors with short detection times (approx. 1 min) [263]. RIfS-based biosensors allow rapid, reagentless detection of proteins [264] and are capable of delivering multiplexed assays [265,266].

## 7. Summary and Future Prospects

The detection of pathogens, drugs, cells and biomarkers is pivotal for the diagnosis of several diseases. Their low amounts in biological fluids present a major hurdle in rapid, reproducible and sensitive quantification. However, optical sensors are now capable of ultrasensititive detection over wide investigation ranges. The fabrication of such sensors is essential for diagnosing diseases at an early stage and thus enables efficient prognosis, treatment and monitoring. We have highlighted the extraordinary performance of optical sensors in this review with the help of some prominent optical phenomena such as surface plasmon resonance, evanescent wave, surface enhanced Raman scattering, etc. The performance of these sensors can be tuned using nanomaterials of varying shapes and sizes facilitating the development of facile, rapid and stable detection platforms for clinical applications such as diagnostics and imaging. Furthermore, high-affinity optical sensors exhibit versatility with different receptors such as antibodies, aptamers, DNA, MIPs, peptides and other biomolecules thereby encouraging the development of various types of assay platforms such as immunosensors, aptasensors, genosensors, cytosensors, and many others. 

The latest trends in optical sensors involve the incorporation of nanostructures substrates to the optical system as well as conjugating different sensing mechanisms to obtain multiple responses from the same sensor. The high cost and low stability of nanomaterials coupled with complicated development strategies have hindered the commercialisation of many optical sensors. Therefore, approaches for the replacement of conventional sensing techniques such as ELISA or PCR with modern optical biosensors are under development. 

Of all research works on optical sensors for healthcare diagnostics, 58% have been reported in the previous half decade underlining the growing interest in such robust detection platforms. The use of optical sensors in conjunction with novel pathogens and biomarkers is projected to increase considerably in the coming years. The facile, lucrative and rapid operational techniques presented by optical biosensors are expected to replace conventional complex and expensive diagnostic tools in the future.
